# Robust adversarial uncertainty quantification for deep learning fine-tuning

**DOI:** 10.1007/s11227-023-05087-5

**Published:** 2023-02-25

**Authors:** Usman Ahmed, Jerry Chun-Wei Lin

**Affiliations:** Department of Computer Science, Electrical Engineering and Mathematical Sciences, Western Norway University of Applied Sciences, 5063 Bergen, Norway

**Keywords:** Adversarial training, Deep learning, Evolution computation

## Abstract

This paper proposes a deep learning model that is robust and capable of handling highly uncertain inputs. The model is divided into three phases: creating a dataset, creating a neural network based on the dataset, and retraining the neural network to handle unpredictable inputs. The model utilizes entropy values and a non-dominant sorting algorithm to identify the candidate with the highest entropy value from the dataset. This is followed by merging the training set with adversarial samples, where a mini-batch of the merged dataset is used to update the dense network parameters. This method can improve the performance of machine learning models, categorization of radiographic images, risk of misdiagnosis in medical imaging, and accuracy of medical diagnoses. To evaluate the efficacy of the proposed model, two datasets, MNIST and COVID, were used with pixel values and without transfer learning. The results showed an increase of accuracy from 0.85 to 0.88 for MNIST and from 0.83 to 0.85 for COVID, which suggests that the model successfully classified images from both datasets without using transfer learning techniques.

## Introduction

Deep learning (DL) methods can also successfully learn from noise or contain errors [1]. This contrasts traditional machine learning methods, which often fail on noisy or erroneous data. Recent advances have been made in image analysis, speech recognition, and game strategy. A deep neural network can perform as well as or better than a human in some situations [2–4].

One of the biggest challenges for DL is the need for more interpretability. Neural networks are black boxes, meaning it is often difficult to understand how they arrive at their predictions [5]. This can be a problem when it comes to making decisions that can significantly impact people’s lives, such as medical diagnoses [6]. Another challenge is that DL requires much data. This can be a problem for areas where data is scarce, as in many areas of science and engineering. Despite these challenges, DL is a promising area of AI research and will likely continue to be used in many different applications [7]. DL is particularly useful in computer vision applications such as surveillance and biometric verification systems because of its ability to identify underlying patterns and make predictions. Generative Adversarial Networks (GANs) offer an additional advantage in that they can generate artificial data that is difficult to distinguish from real-world data, even to the human eye [8]. However, DL models are vulnerable to adversarial attacks in which small changes to the data can lead to misclassifications, and predictions [9], which can have significant implications for biometric recognition and autonomous vehicles. Therefore, researchers must continue to look for ways to defend against such attacks [10].

Concerning such critical systems, our artificial intelligence models must be free from error. The system is not expected to be omniscient or one hundred percent accurate, but we can ask it to let us know when it is unsure. Then a second check can be performed or the task can be delegated to a human professional. Conventional DL networks, therefore, cannot consider information about uncertainty because they only make point predictions. A distribution over potential classes is sometimes used to convey confidence in the classification questions for each class. However, results may need to be more accurate if the input is unclassifiable and does not correspond to a class. This study addresses EQ improvement issues that generally do not provide new instance generation in uncertainty estimates. Several studies have already been conducted as part of the initiative. The technology is being modified in various ways to improve the performance of networks in prediction or classification and to incorporate some level of confidence into the process.

### Motivation

Uncertainty quantification (UQ) is a growing area in DL that deals with quantifying uncertainty in DL models. UQ is important in DL because it allows us to quantify the degree of uncertainty in our predictions. This is important because it allows us to identify when our predictions are likely wrong and adjust our models accordingly. UQ can be used to improve the transparency of DL models and identify potential problems such as bias and overfitting. By quantifying the model’s output uncertainty, we can better understand the model’s decision-making process and identify potential problems. It also allows us to understand the limitations of our models better and identify areas where further research is needed.

There are two motivations behind the research. First, DL models are often used in applications where errors can have serious consequences, such as medical diagnosis and self-driving cars. Second, DL models are often opaque and complex, making it difficult to understand and rely on their predictions. Transfer learning methods and UQ approaches are two strategies that can be used to improve machine learning performance in medical categorization tasks. UQ techniques use data from a variety of sources to quantify the uncertainty associated with a given prediction, allowing for more accurate results and better decision-making. By integrating transfer learning with UQ techniques, medical classification problems can be predicted more accurately and features extracted more precisely. UQ can help solve both problems by providing a way to measure and characterize the uncertainty of DL models.

### Contributions

The DL model is trained in this study, and the hyperparameters are adjusted to achieve maximum accuracy. A sensitivity analysis is then performed to determine how different input parameters affect the model’s output. The most uncertain instance in the training was selected using an entropy-based method. Using the uncertainty maximization method, we applied the Non-dominated Sorting Genetic Algorithm-II (NSGA-II) method to generate new patterns. Based on the training data, new instances are generated using the following optimization function: the prediction uncertainty should be maximized, and the amount of noise should be minimized. Then the new samples are used to retrain and optimize the model to achieve higher accuracy and reduce the uncertainty in prediction.

1.  For the main contributions, first, we will then identify areas of a dataset that could use further exploration to raise the model's accuracy. Uncertain classes determine what more data or resources are necessary to increase the precision of the model.
2. In addition, we will then recognize areas which can be enhanced in models, ultimately leading to improved predictive performance. By merging these approaches, data analysts can refine their multi-classification models and generate more precise forecasts.

## Related work

UQ in DL involves assimilating uncertainty into the learning procedure. UQ merges the DL approach to estimate the likelihood of a given outcome [11]. By incorporating uncertainty into the learning process, UQ can improve the accuracy of predictions and provide more reliable results [12]. UQ uses various techniques to quantify uncertainty, including Bayesian inference, Monte Carlo sampling, and Bayesian optimization. These techniques allow UQ to account for data variability and model parameters’ uncertainty. This allows UQ to better account for the complexity of the data and make more accurate predictions. UQ can also be used to improve the accuracy of models from DL. By incorporating uncertainty into the learning process, UQ can help reduce the risk of overfitting and improve the accuracy of predictions. UQ can also be used to improve the interpretability of DL models, which is essential for understanding and explaining the results of a model [13]. Overall, UQ is a powerful tool for improving the accuracy and interpretability of DL models. Incorporating uncertainty into the learning process can reduce the risk of overfitting and improve the accuracy of predictions. UQ can also be used to improve the interpretability of DL models, which is essential for understanding and explaining the results of a model [14].

As explained earlier, another paper introduces a new class of momentum-based iterative algorithms that can boost adversarial attacks on deep neural networks [15]. Since these attacks can have severe consequences, they are often used to measure the resilience of a DL model. A momentum term is incorporated into the iterative process, which allows attackers to bypass bad local maxima and generate more effective adversarial examples during the iteration process. This paper also describes how the proposed method was used to win first place in the Non-targeted Adversarial Attack and Targeted Adversarial Attack competitions at NIPS 2017.

A novel attack scheme for semi-supervised learning (SSL) systems is presented that uses malicious unlabeled data to inject a backdoor into the model [16]. A robust perturbation generator is used to create poisoned data, and a novel contrastive data poisoning strategy is developed to reduce the negative impact of trigger patterns on model accuracy and increase the success rate of the attack. Experiments were conducted on the CIFAR10 and CIFAR100 datasets to evaluate the effectiveness and crypticity of the proposed method.

Person search is a challenging task that involves locating and identifying a wanted person in real, untrimmed images. Current approaches use two-stage detectors such as Faster-RCNN, which incur high computational overhead [17]. The Feature-Aligned Person Search Network (AlignPS) is an anchoring-free system for solving this task. AlignPS counters misalignments between different scales, regions, and tasks by proposing a feature aggregation module that generates more discriminative and robust feature embeddings. This approach improves the baseline anchor-free model at CUHK-SYSU by more than 20% in mAP and outperforms state-of-the-art two-stage methods with higher speed.

Autism spectrum disorder (ASD) is a developmental disorder that affects interaction and communication with others and results in repetitive behaviours. This paper presents a new video dataset and a DL network, One Glimpse Early ASD Detection (O-GAD), designed to detect ASD-typical actions and repetitive behaviours in videos of arbitrary length [18]. Experiments have shown that O-GAD outperforms existing methods in terms of mAP, and extensive ablation experiments have been conducted to validate the effectiveness and rationality of the network structure.

A unified framework for action recognition, called Event Adaptive Network, is proposed to model spatio-temporal information in videos for better action recognition. It has two main components: (1) dynamically scaled spatio-temporal kernels suitable for events of different sizes, and (2) a transformer that selects a few foreground objects and evaluates their interactions, resulting in a sparse paradigm. In addition, a Latent Motion Code module is proposed to exploit short-term motion within local segments. Experiments on large-scale datasets show that the proposed model achieves state-of-the-art or competitive results with low FLOPs.

A novel approach to motion representation in computer vision tasks [19] is proposed. The approach uses unlabeled video data to learn an explicit motion representation optimized for the semantic distribution of moving objects. A coarse-to-fine paradigm is used, where low-resolution motion maps are first decoded from the spatio-temporal features of the video, followed by adaptive upsampling of the maps to full resolution with semantic cues. The proposed context-driven upsampling layer learns the upsampling parameters based on the spatial context of the video objects. The effectiveness of this motion representation method is demonstrated through experiments with action recognition tasks, which show that it outperforms existing methods.

Bicubic downscaling is a popular technique to reduce video memory and speed up downstream processing [20]. However, the inverse upscaling step is difficult, and the downscaled video often causes performance problems in downstream tasks. In this work, a self-conditioned probabilistic video rescaling method is proposed that simultaneously learns the downscaling and upscaling processes. During training, the authors reduce the information lost during downscaling by maximizing its probability, using the strong spatio-temporal prior information in the downscaled video. After optimization, the downscaled video contains more meaningful information that helps in upscaling and downstream tasks such as video action recognition. The method is also extended to a lossy video compression system using a gradient estimator for non-differential industrial lossy codecs for end-to-end training. The results show the superiority and effectiveness of this approach in video rescaling, video compression, and action recognition.

Dual Generative Adversarial Active Learning (DGAAL) is a novel active learning method that combines pool-based and synthesis-based approaches to reduce annotation costs while maintaining good model performance [21]. It uses two Generative Adversarial Networks (GANs) consisting of a generator and two discriminators. The first GAN is used to learn the data representation, while the second is used to generate samples. The two GANs work together to allow the generated samples to be included in the sampling process and the discriminator used for sampling to evolve along with them. This allows the use of samples with rich information in the later stages of sampling, mitigating the problem of insufficient information for sample selection in the pool-based method. DGAAL was tested extensively on three datasets. The results showed that it has certain advantages over existing methods in terms of model performance and significantly reduces annotation costs.

A new approach to reduce the cost associated with classification errors in DL classification tasks [14]. This approach combines two main approaches: (1) quantifying the uncertainty of a classifier’s predictions to reduce the probability of responding to erroneous predictions, and (2) a novel method of training the classifier to erroneous bias classifications toward less risky categories [14]. To achieve this, the authors extend Deep evidential learning to include pignistic probabilities, which are used to quantify the uncertainty of classification predictions and to model rational decision-making under uncertainty. The performance of the approach is evaluated on several image classification tasks. Results show that it enables the incorporation of misclassification costs when deep training classifiers, accurately quantify the uncertainty of classification predictions and simultaneously learns how to make classification decisions to minimize the expected cost of classification errors.

An unsupervised cross-domain ReID method based on median stable clustering (MSC) and global distance classification (GDC) is proposed [22]. The MSC method uses a measurement method that considers the similarity between clusters, the number of samples in a cluster, and the combined similarity within a cluster. At the same time, GDC can separate the distance distribution of positive and negative sample pairs in a global framework, unlike the method based on triple loss. In addition, to reduce the influence of parameters on the performance of source memory reconsolidation, a dynamic memory reconsolidation (DMR) method was developed. To evaluate the effectiveness of the proposed method, experiments are conducted on three large datasets (Market-1501, DukeMTMC-reID, and MSMT17). The experimental results show that the proposed method MSC-GDC outperforms state-of-the-art methods in terms of performance.

UQ methods are widely used to solve optimization and decision problems in various practical applications. It reviews the use of UQ methods in DL and reinforcement learning [11]. It discusses the various UQ methods used in DL for computer vision (e.g. self-driving cars and object detection ), image processing (e.g. image restoration ), medical image analysis (e.g. medical image classification and segmentation), and natural language processing (e.g. text classification, social media text, and recidivism risk assessment). In addition, applications of UQ methods to reinforcement learning and related research challenges and directions will be discussed.

There are still many challenges to be overcome before DL can be used for safety-critical applications [23]. DL networks are not fully explained in terms of how and why they work, which is a significant obstacle. This means that researchers, developers, and users need to understand how the DL network works and why it makes its decisions. With the increasing use of DL in safety-critical applications, such as autonomous vehicles, this issue has become increasingly important [24]. Trusting DL networks in these safety-critical applications is demanding without understanding how and why they work. To address this problem, researchers are exploring various explanatory methods, including saliency and counterfactuality, to make DL networks more transparent and explainable [25, 26]. Two issues are discussed in the context of using deep neural networks (DNNs): explaining neural network functions to the user and the sensitivity of DNNs to changes in inputs. In medical applications, it is crucial to explain the neural network function to the user so that the user can understand what the neural network does and how it makes decisions. In addition, neural networks are susceptible to changes in the input distribution. When choosing an appropriate dataset for a particular task, it is essential to consider the sensitivity of DNNs trained on the ImageNet dataset [27]. They are susceptible to changes in the input data, which can lead to poor results when a model trained on one dataset is used to classify images from a different domain. This is often referred to as domain adaptation and is a critical problem that must be addressed if DNNs are to be used in real-world applications.

Furthermore, when using neural networks, it is often necessary to explain the function of the network to the user, mainly when it is used in medical applications. Finally, DNNs have difficulty dealing with noisy data and high uncertainty [28]. Therefore, it is crucial to train a DNN model that can work effectively on different types of data and under real-world conditions.

Testing models before they are deployed in new environments is essential to ensure accurate results [29]. Traditional testing methods, such as statistical testing, modelling, and validation, often do not provide a robust test set that can effectively cover all possible scenarios [30]. ML models must follow software testing methods to ensure successful industrial applications. There are three software testing methods: White-box testing, black-box testing, and grey-box testing [31]. Grey-box testing uses elements from both white-box and black-box testing. Here, the system’s internal structure is used to generate test data, and the environment is used to validate the test. In summary, grey-box testing is a comprehensive approach to testing ML models before deployment because it considers both the internal structure of the system and the environment in which the system operates.

A novel approach to modelling the predictive uncertainty of deterministic neural networks by explicitly incorporating the theory of subjective logic [32]. By placing a Dirichlet distribution on the class probabilities, this method treats the predictions of a neural network as subjective opinions and learns the function that gathers the evidence leading to these opinions from the data by a deterministic neural network. The preliminary analysis results have shown that this new loss function can improve uncertainty estimation. It also effectively detects queries outside the distribution and has higher resilience to negative perturbations. This method is an orthogonal approach to Bayesian neural networks, which derive the uncertainty of the prediction only indirectly through the weight uncertainties.

Recent advances in digital pathology and DL have enabled robust disease classification, better diagnosis, and improved prognosis. However, in the real world, weak image-level labels from pathology reports pose a challenge to improving the performance of DL models. To address this challenge, the Uncertainty-Aware Sampling Framework (UASF) [33] is an importance-based sampling framework for robust histopathology image analysis. UASF was tested on a highly heterogeneous subtype of soft tissue sarcoma and demonstrated improved accuracy compared with baseline models. UASF is effective in sampling the most relevant tiles and can be used to improve the accuracy of DL models.

Chest radiographs can reliably and rapidly detect coronavirus disease (COVID-19) [34]. The authors developed a large X-ray dataset (COVQU) consisting of 18,479 CXR images, including standard images, non-COVID lung infections, COVID-19 CXR images, and the associated ground truth lung masks [34]. Five different image enhancement techniques were used to investigate the effects of image enhancement techniques on COVID-19 detection. A novel U-network model was proposed for lung segmentation, and six different pre-trained convolutional neural networks (CNNs) were used to detect COVID-19 from simple and segmented CXR lung images. The study results show that the gamma correction-based enhancement technique outperformed the other techniques in detecting COVID-19 from simple and segmented lung image CT. While the classification performance of plain CTXR images was slightly better than that of segmented lung CTXR images, the network performance reliability of segmented lung CTXR images was significantly better. For segmented lung images, accuracy, precision, sensitivity, F1 score, and specificity were 95.11 percent, 95.55 percent, 95.56 percent, 95.56 percent, 95.53 percent, and 95.59 percent, respectively. The proposed method, with its highly reliable and comparable performance, is believed to improve the rapid and robust detection of COVID-19 from chest X-ray images.

UQ is used to quantify and reduce uncertainty in various computational and practical contexts [35]. As more training data and complex models can reduce the probability of wrong decisions, it becomes infeasible to consider all possible cases. Therefore, a model should be able to demonstrate its reliability by successfully inferring knowledge from unknown circumstances and emphasizing the result [36]. UQ is used to investigate what exactly it is that the model does not know because it is known that there are unknown factors that the model does not know from training.

### Source of uncertainty

There are a variety of uncertainties associated with practical applications and mathematical models [11].

1. **Parameter uncertainty:** Parameter uncertainty is an input uncertainty that arises when the parameters used in a model or process are not known. It is one of the three major sources of modelling uncertainty [28]. Parameter uncertainty can have various sources, such as measurement errors, a lack of knowledge or understanding of a system, and even randomness. Fortunately, there are various methods to address parameter uncertainty, including sensitivity analysis, Monte Carlo simulations, and expert judgment. It is critical to consider parameter uncertainty during risk assessment and decision-making, as it can significantly affect the performance of a model.
2. **Model inadequacy:** If a model does not accurately capture all relevant aspects of a problem, the model is inadequate. It can manifest itself in various ways, including inaccurate predictions, overfitting, or failure to capture all nuances [37]. Several components can lead to a model being inadequate, such as data leaks, overfitting, or a lack of data. It is critical to mitigate model inadequacy through data cleansing, data preprocessing, and feature selection in order to guarantee precise predictions and a successful model. Regular inspections and examinations of the model can help to uncover potential inadequacies and resolve them in a timely manner.
3. **Residual variability:** is the amount of variability that remains after all other known factors or variables have been controlled [38]. There are a number of reasons for this, such as random errors or unknown variables that have not been taken into consideration. Residual variability is an integral component of statistical analysis, as it helps in recognizing sources of unexplainable differences in populations and groups. Furthermore, it can be used to assess the relative importance of various factors or variables in determining the observed variability of a given data set.
4. **Parametric variability:** is a type of variability caused by changes in the parameters of a system. Parametric variability is a term used to describe variability in the parameters of a system. Intentional or unintentional changes can result in a wide range of outcomes due to these changes [37]. Parametric variability refers to the alterations in the behaviour of a system when its parameters are modified. This type of variability can be manifested through changes in the design, components, environment, operation, or inputs of the system. Depending on the parameters used, parametric variability can have both positive and negative effects on the performance of a system or process. Through parametric variability, one can analyse the potential outcomes of a system or process and identify areas of improvement in order to optimize the system's performance and behaviour.
5. **Observation error:** is the difference between the observed value of a measurement and its actual value. This type of error occurs when the person making the measurement is not precise or accurate enough in their measurements to account for it [39]. For instance, a weather station can experience an observational error if the temperature is off by one degree Celsius. This can be due to instrument failure, incorrect calibration, or human error. To minimize these errors, it is important to take multiple measurements and compute the average or median of the results. Knowing the sources of observational errors is essential as it can lead to wrong conclusions and decisions.
6. **Code uncertainty: ** Code uncertainty is a consequence of numerical approximations and errors during the training process, which means that the results of a machine learning model are not guaranteed to be accurate [40]. Numerical approximations occur when real numbers are converted to binary numbers and vice versa, introducing various errors during the training process. This code uncertainty can result in inaccurate outcomes and should be taken into account when striving to achieve the best possible results. Factors such as misconfigured models, noise in the data, or other variables can all contribute to this. To reduce the effects of these errors, it is essential to carefully consider and monitor the model throughout the training process.

### Uncertainty types

1. **Epistemic uncertainty:** is a type of uncertainty that is generated internally within a model, instead of originating from external sources. It is the result of the restricted knowledge and data available to the system, such as when the model fails to consider certain factors or measurements are not precise [41]. The level of uncertainty can be minimized by increasing the number of training samples. This is because the model will be able to better incorporate the components that it previously disregarded, leading to more accurate measurements.
2. **Aleatoric uncertainty:** is a type of uncertainty that exists within a given dataset, which encompasses both statistical uncertainty and data uncertainty. Statistical uncertainty is derived from the fact that the dataset is a sample of a larger population, and thus may not accurately reflect the population as a whole. Data uncertainty is based on the fact that data can be incomplete or biased, leading to inaccurate conclusions. Both of these forms of uncertainty contribute to aleatoric uncertainty, and can result in significant discrepancies between the actual data and the conclusions drawn from it [41].

This section introduces the concept of uncertainty, its sources, and its various categories. We will explore how uncertainty can be quantified to better understand and address potential risks and opportunities in an uncertain environment. Whenever outcomes are uncertain, quantifying uncertainty is a critical step in decision-making. To quantify uncertainty, we use statistical and probabilistic methods such as Monte Carlo simulations or Bayesian networks, which allow us to make more informed decisions.

### Uncertainty quantification approaches

This section discusses different approaches for estimating uncertainty in experiments.

1. **Monte Carlo dropout:** is a technique that reduces overfitting in deep neural networks by using multiple iterations of a stochastic process to approximate the expected value of the model with a single network. As part of the process, a random number of nodes (or neurons) are removed from the network, and the network output is then computed based on the test data [11]. A Monte Carlo estimate of the expected value of the model is obtained by repeating this process for multiple iterations (or “dropouts”). In deep neural networks, Monte Carlo dropout effectively reduces overfitting because it reduces the model’s dependence on specific neurons and encourages the network to learn from all of its features. This technique has led to significantly better generalization results than other techniques, such as dropout and early stopping. It has been shown that the Monte Carlo dropout approach can reduce overfitting in deep neural networks by reducing the number of nodes in the network. To produce a Monte Carlo estimate of the expected value of the model, randomly selected nodes are “dropped out” of the network, and this process is repeated. This technique has yielded better generalization performance than other techniques, making it an effective means of reducing overfitting in deep neural networks.
2. **Deep ensembles:** An ensemble approach to UQ identifies the uncertainty associated with predictions by analysing the same data set using multiple machine learning models with different parameters [11]. This approach is used to identify the uncertainty associated with the predictions to improve their accuracy. The method combines the predictions of several models and then analyses the variance between them to determine the degree of uncertainty associated with each prediction. It is possible to improve the accuracy of the predictions by measuring the variance and then combining them. This approach can be used to understand better the uncertainty associated with the predictions. This can reduce the risks associated with decisions based on the predictions.
3. **Ensemble MC dropout approach** The ensemble MC dropout approach combines the two techniques to create a powerful technique for UQ [42]. Combining the two techniques, the ensemble MC dropout approach can provide better uncertainty estimates than either. In particular, it has lower bias and higher predictive accuracy than either technique alone. It also has a lower computational cost than deep ensembles. This makes it an attractive choice for UQ in DL.

### Uncertainty computation measures

This section discusses computation methods based on UQ that can be used for understanding the effects of uncertainty.

1. Prediction variance measures the uncertainty in a prediction or forecast. It is a measure of how much the predicted values deviate from the mean of the observed data. It is the degree to which predictions or forecasts are uncertain. In predictive analysis, forecast variance is often used to measure the accuracy of a model. It is an important tool for analysing the predictive quality [43]. When calculating the uncertainty of a prediction or forecast, it is important to consider the prediction variance. The larger the variance, the less accurate the predictions. A large variance in the prediction indicates a high degree of uncertainty. Conversely, a low prediction variance indicates a low degree of uncertainty in the prediction. The variance of the prediction can be affected by several factors, such as the type of model used, the training data used, the underlying assumptions, and the number of parameters in the model. In addition, the variance of the prediction can also be affected by the method used to estimate the uncertainty of the prediction. Therefore, it is important to consider the prediction variance when calculating the uncertainty of a prediction or forecast.
2. The entropy of an information system is a measure of its disorder or randomness, especially in machine learning problems (ML) [44]. It is a measure of the system’s unpredictability and can be used in ML to detect patterns in the data and make decisions. Entropy can be calculated differently depending on the nature of the machine learning problem and the available data. It is a fundamental concept in machine learning because it allows us to evaluate the accuracy of our model’s predictions. It is used to measure the uncertainty of a model, which is essential for decision-making. If a model’s predictions have high entropy, it is uncertain and can be improved. If a model has low entropy, it means that the model is more confident in its predictions and can be trusted. In addition, entropy can be used to determine which model is most likely to produce the best results. We can determine which model will produce the best results by measuring entropy. Our models will likely be optimized if we can evaluate the quality of our predictions. Therefore, entropy is a crucial concept in machine learning because it allows us to choose the best model for a given problem based on the quality of its predictions. If we understand entropy, we can make better decisions and develop better machine learning models.

## Methodology

**Fig. 1:**
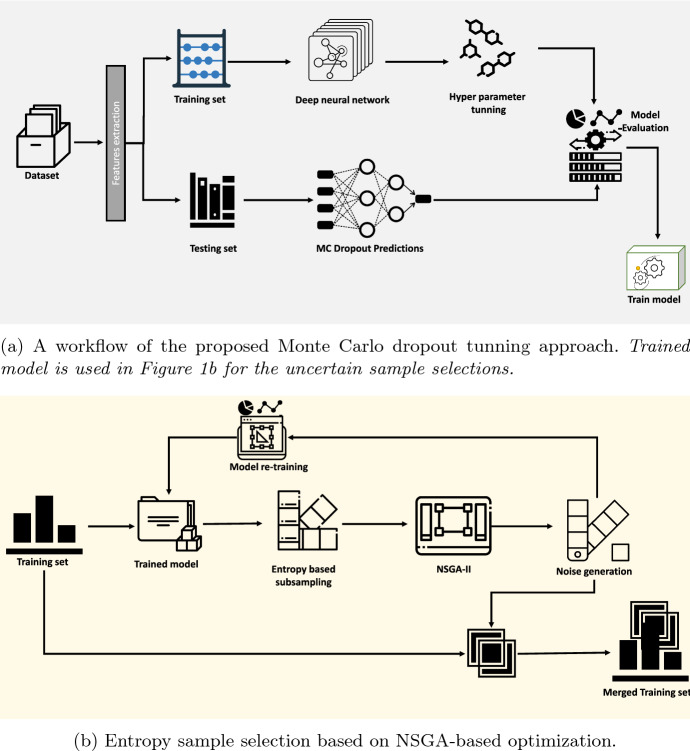
Uncertainty sample selection and optimization for the hyper-tuning

This work aims to develop a robust DL model that can generate highly uncertain inputs, as mentioned in Fig. 1a and b. This research seeks to devise a robust deep learning (DL) model capable of dealing with highly uncertain inputs. The proposed approach involves three phases. In the first phase, a dataset is generated. In the second phase, a neural network is constructed based on the dataset of failures to make accurate predictions. Lastly, the model is fine-tuned in the third phase with inputs that are highly uncertain. By incorporating dropout-based neural networks, the model is expected to be more resilient to input changes and more tolerant to erroneous inputs, thus resulting in higher prediction accuracy.

As mentioned in Fig. 1a and b, the proposed model optimizes data by introducing uncertainty. It takes a dataset as input and returns an optimized model. The algorithm begins by extracting features from the dataset using Algorithm 3, and then divides the dataset into a training and testing set (Algorithm 1, Lines 3–4). A dense network is then initialized and a mini-batch of training examples is selected. The dense network parameters are then updated using backpropagation (Algorithm 1, Lines 5–9). Entropy values for all samples in the dataset are calculated and the sample with the highest entropy value is identified as a candidate (Algorithm 1, Lines 11–24). The candidate entropy value is subtracted from the total entropy value and the candidate is added to a set. Data density estimation can be particularly challenging when dealing with high-dimensional image data. To this end, a Monte Carlo method is employed to train a deep learning model, and the class probabilities of the model are used to identify samples with high uncertainty. We provide explicit procedures for calculating entropy. Entropy is a measure of uncertainty and is calculated as follows:

1 $$\begin{aligned} \textrm{Entropy} = - \sum _{i=1}^{n} p_i \mathrm{\log }_2 p_i \end{aligned}$$

where $p_i$ is the probability score for the classification. For example, if the sample values indicate that the probability of class A is 0.7 and the probability of class B is 0.3, then the entropy would be calculated as follows:

$$\begin{aligned} \textrm{Entropy}= & {} - (0.7 *\textrm{log}\; 2 (0.7)) - (0.3 * \textrm{log}\; 2 (0.3))\\= & {} - (0.48) - (0.46)\\= & {} -0.94 \end{aligned}$$

High entropy signifies that the probability values for the classification are evenly distributed, thus the uncertainty is high. Conversely, a low entropy indicates that the probability values for the classification are not uniformly dispersed and therefore the uncertainty is low. This process is repeated until the desired number of candidates are selected. Afterward, the algorithm performs a non-dominated sorting algorithm 2 on the chosen candidates and combines the training set with the generated adversarial samples (Algorithm 1, lines 24–26). Subsequently, the algorithm mini-samples the merged dataset and adjusts the dense network parameters via backpropagation (Algorithm 1, lines 26–30). Finally, the algorithm returns the optimized dense network.

**Figure:**
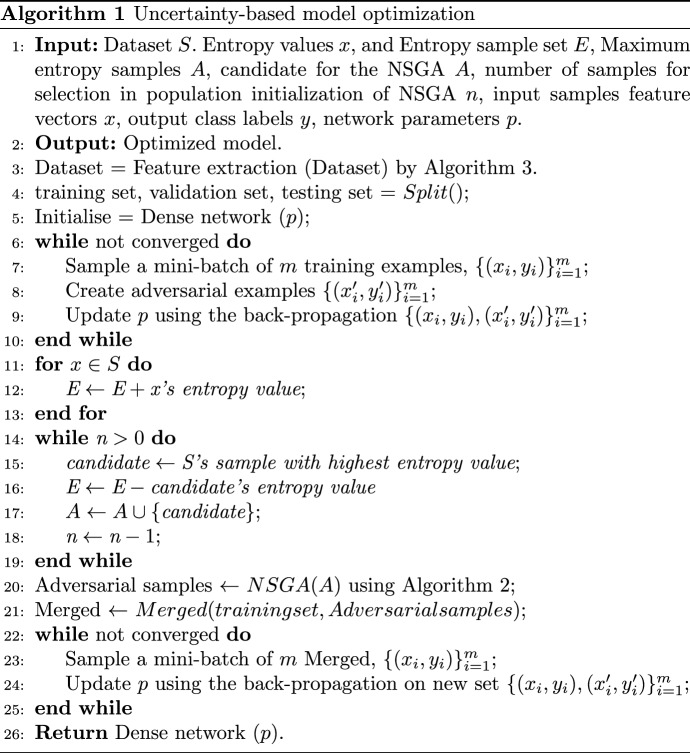


### Using the non-dominated sorting genetic algorithm to create test inputs

The Non-dominated Sorting Genetic Algorithm-II (NSGA-II) is an evolutionary algorithm designed to address multi-objective optimization problems. This type of algorithm uses a genetic-based approach to identify solutions that are not dominated by other solutions in terms of objectives. NSGA-II applies a sorting technique to identify such solutions, helping to optimize the overall performance of the system [45, 46]. NSGA-II is a type of genetic algorithm that not only follows the general outline of a genetic algorithm but also makes changes to the selection process for mating and survival. In this algorithm, individuals are selected frontally, meaning that if not all individuals can survive, a front must be divided. This dividing front selects solutions by calculating the displacement distance. The advantages of NSGA-II include its ability to handle mixed constraints, elitism, a very low likelihood of premature convergence to local optima, and computational efficiency. Pymoo is used to implement the algorithm and the output of the NSGA-II algorithm is the set of non-dominated solutions. This algorithm requires inputs such as the size of the population, the number of generations, the objective function, and the lower and upper bounds of the decision variables.

**Figure:**
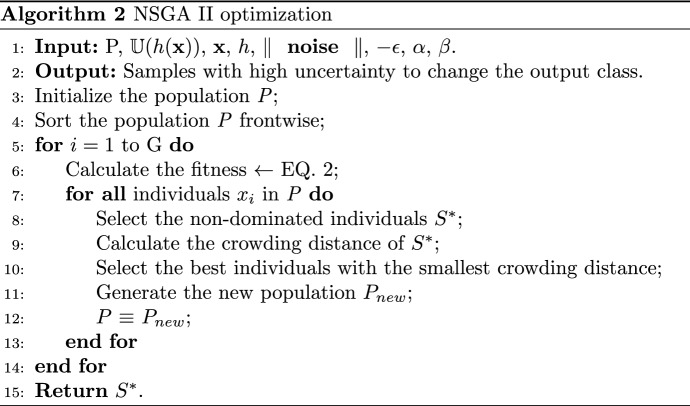

This algorithm is used to identify samples with high uncertainty in a given dataset. The input to this algorithm is the dataset *X*, $\alpha$, $\beta$, and the noise parameter given in the Eq. 2. (Algorithm 2, Line 1). (e.g., The output of Algorithm 2 is a set of samples with high uncertainty. Population-based optimization algorithms typically begin by randomly initializing the population (P) with a set of individuals, or solutions, representing the initial population of the optimization problem. Each individual in the population consists of a set of parameters (e.g., weights in a neural network) that can be used to obtain a particular solution. Randomly initializing the population with these parameters can be a great starting point for the optimization process and can help the algorithm to quickly converge to an optimal solution. This algorithm implements the Non-Dominated Sorting Genetic Algorithm (NSGA) II, an evolutionary algorithm. The algorithm takes a population of individuals (P), an uncertainty function (U), an input vector (x), a scoring function (h), and a set of parameters (noise, epsilon, alpha, and beta). It starts by initializing the population P and sorting it forward. Then, it iterates through a number of generations (G) where it calculates the fitness of each individual using the noise function and selects the non-dominant individuals in the population. These non-dominant individuals are selected based on their displacement distance, and the best individuals with the smallest displacement distance are chosen. The new population (P) is derived from these best individuals. Finally, the algorithm returns the set of non-dominant individuals (S*).

### Objective function

In this study, we used the optimization method mentioned in Eq. 2. This technique employs an optimization process, as outlined in Algorithm 2, to generate complex inputs or inputs with a high level of uncertainty. The objective of this optimization process is to maximize the uncertainty of the input prediction, which is achieved by increasing the uncertainty, reducing the perturbation amount, and increasing the number of incorrect predictions [47]. The goal is achieved by combining two hyperparameters, alpha and beta, to find the optimal solution. The result is new instances based on the existing training data.

2 $$\begin{aligned} {\textbf { noise }} \equiv \underset{ \text{ noise } }{\arg \min }\left( \frac{\alpha }{\mathbb {U}(h(\textbf{x}))}+\Vert \text{ noise } \Vert +\beta \cdot \mathbb {1}(y \equiv \hat{y})\right) , \Vert {\textbf { noise }} \Vert <\epsilon \end{aligned}$$

1. $\mathbb {U}(h(\textbf{x}))$ is the prediction uncertainty
2. $\mathbf {-} \textbf{x}$ is the input instance
3. *h* is the DL model
4. $\Vert$ **noise** $\Vert$ is the norm (i.e. magnitude) value of the noise.
5. $-\epsilon$ is the noise budget (i.e. the maximum distance between $\textbf{x}$ and $\textbf{x}+$ noise )
6. $$-\textbf{x} \in \mathbb {R}^m$$ and noise $$\in \mathbb {R}^m$$
7. noise has the same number of columns as the input instance $\textbf{x}$
8. $\alpha$ is the multiplication factor of UQ metrics value
9. $\beta$ is the multiplication factor of the number of incorrect predictions

The describes the ideal noise would have properties that are both desirable and useful as follows:

- Maximizing predictive uncertainty is a strategy for improving the confidence and accuracy of predictions. This involves the creation of a model that takes into account multiple sources of uncertainty, such as data bias, model complexity, and environmental conditions. Additionally, the model should be capable of recognizing and eliminating uncertainty to improve the precision of the predictions. The aim is to guarantee that the model does not overpredict or underpredict but provides the most accurate predictions possible. This approach can be employed to enhance the accuracy of machine learning models, making them more dependable and trustworthy.: $\frac{\epsilon }{\mathbb {U}(\textbf{x})}$
- Reducing the noise magnitude (norm value) is a strategy used to decrease the impact of noise on a system, thereby diminishing the effects of uncertainty on the optimization process. This approach helps to optimize the system by decreasing the variability of the input data, thus minimizing the randomness' influence on the process. The objective is to reduce the general uncertainty created by noise, so that the optimization process yields more precise and dependable results as: $\Vert$ **noise** $\Vert$
- Optimization is a technique used to enhance the accuracy of machine learning models in the presence of uncertainty. This technique involves changing the prediction class to decrease the uncertainty associated with the model's forecasts. This approach can help improve the accuracy of the model and make its predictions more dependable. It can also be used to reduce the risk of overfitting the model, which can result in inadequate generalization performance as: 1$(y\equiv \hat{y})$

### Feature extraction

This method resizes an image and extracts its features as described in Algorithm 3. This method begins with an image and a size parameter N. An empty matrix of N x N is then created. The average of the pixels at position (x, y) for each pixel in the image is calculated and stored in the new matrix. Following the resizing of all pixels in the image, features are then extracted from the new image. To do this, each pixel value from the new image is appended to a set of features. Finally, the algorithm returns the set of features.

**Figure:**
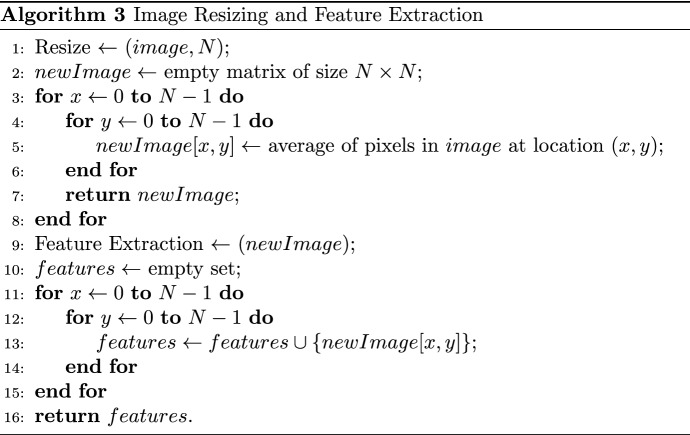

This paper proposes a method for improving the robustness of Deep Learning (DL) models against adversarial attacks. The technique involves perturbing the training set with a small amount of noise generated via the NSGA-II optimization-based perturbation method. This approach has the potential to enhance the resistance of DL models to adversarial attacks, thereby increasing their security. (Algorithms 1 and 2) to increase the uncertainty of the test input for the DL model. Algorithm 1 is presented to take the dataset and output the robust model. UQ is an essential ingredient in ensuring the accuracy of results. Without quantifying the uncertainty associated with a data set, researchers may draw incorrect conclusions from the data. To adequately quantify the uncertainty of a dataset, it is important to consider the quality of the data and the potential for collecting additional data or conducting additional analyses. The proposed method for quantifying the uncertainty of a dataset is robust enough to handle highly uncertain inputs. The model is trained to identify and classify data using entropy values and a non-dominant sorting algorithm to identify the candidate with the highest entropy value from the dataset. In addition, the model is retrained to handle unpredictable inputs by merging the training set with adversarial samples and then updating the dense network parameters with a mini-batch of the merged dataset. This indicates that the proposed method can accurately quantify the uncertainty of a dataset even when the dataset does not contain significant uncertainty. The proposed method is a valuable tool for researchers when evaluating the accuracy of their data and results, as it can help reduce the impact of uncertainty on the optimization process by minimizing the amount of noise in the system.

## Experimental results

This paper investigates the use of deep learning (DL) and hyperparameter tuning to improve the classification accuracy of medical imaging. An entropy-based method was used to identify the most uncertain instance in the training dataset, followed by a sensitivity analysis which determined how different input parameters affected the model's results. The NSGA-II entropy-based sampling method was then employed to generate new samples to maximize uncertainty and minimize noise values. The methodology was tested on the COVID Radiography Database, yielding promising results. The findings suggest that uncertainty quantification (UQ) can be used to improve the performance of machine learning models, improve the classification of radiographs, and assess the risk of misdiagnosis in medical images, among other applications. This study thus provides evidence that UQ can be a useful tool for optimizing healthcare systems.

### Experimental setup

Python is a powerful programming language that can be used to define models and visualize analysis. Numerous packages are available for this purpose, including TensorFlow, Uncertainty Wizard, Pandas, NumPy, Sci-Kit Learn, SciPy, Keras, Seaborn, Pymoo, CleverHans, Tqdm, and Matplotlib. These packages can build and train models, perform statistical analysis, create data visualizations, and more. With these packages, it is possible to uncover trends, patterns, and insights in data that would be easier to discover by using Python.

Convolutional Neural Networks (CNNs) are a state-of-the-art model for image classification tasks, as they are capable of capturing complicated features such as shapes, edges, and patterns from images [48]. In this paper, we present a model for supervised learning tasks that consists of a convolutional layer and three other dense layers. The convolutional layer has a kernel size of 3x3, a stride of 2, and a padding type of ’same’. A dropout layer with a dropout rate of 0.09 is used to avoid overfitting. The output of the convolutional layer is fed into a first dense layer with 2000 neurons and a relu activation function. This is followed by a second dropout layer with a dropout rate of 0.1. The output of this layer is fed into the second dense layer with 1000 neurons and a relu activation function. A third dropout layer with a dropout rate of 0.09 is followed by the third dense layer with 2000 neurons and a relu activation function. Finally, the output is fed into the output layer with 1000 neurons and a softmax activation function. The model is trained with the Adam optimizer with the categorical cross-entropy loss function and early recalls enabled. The model is trained for a specified number of epochs until the desired accuracy is achieved [48].

This type of DL model is a stochastic sequential Keras model with multiple layers, including three dense layers with 2000, 1000, 2000, 2000, 1000 neurons, respectively, and three dropout layers with 0.09, 0.1, 0.09, 0.1 dropouts to avoid overfitting. The model uses ReLu activations for the input and hidden layers and a softmax activation for the final output layer. To train the model, the Adam optimizer with the categorical cross-entropy loss function is used, and the model is run and trained with early callbacks activated. Table 1 shows the settings of a device, including its architecture, base clock, and memory. The device has an Intel Core i7 (10th generation) 10750 H processor with a base clock of 2.6 GHz and 16 GB of RAM. It also has an NVIDIA GeForce RTX 2070 GPU with a base clock of 993 MHz and 8 GB of RAM. The details of the software environment are listed in Table 1.

**Table 1:** Environment hardware setup

Device	CPU	GPU
Architecture	Intel Core i7 (10th generation) 10750 H	NVIDIA GeForce RTX 2070
Base clock	2.6 GHzz	993 MHz
Memory	16 GB	8 GB

### Dataset

Transfer learning can be a powerful tool to improve the accuracy of models when the target dataset is small or related to the source dataset [49]. For example, ImageNet, a large-scale computer vision dataset, can be used to improve the accuracy of models trained on medical images. However, it is important to consider whether transfer learning is beneficial for a particular task. In cases where the target dataset is unrelated to the source dataset, or where the target dataset is too small to be used effectively for transfer learning, transfer learning may not be beneficial. The case of COVID is an example of a case where transfer learning is not beneficial because the pre-trained model may detect features and patterns in the data that are only obvious to those who already know the data. To train a model from scratch and ensure that it is generalized and robust, it is important to identify areas of the dataset that may require further investigation [50]. This can be done by identifying the most uncertain samples that are the most difficult for the model to classify. Focusing on these samples helps identify areas where additional data or resources are needed to improve the accuracy of the model. Therefore, we do not use the pre-training and training of the model from scratch to test the proposed approach under uncertain conditions.

We use two datasets, MINIST and COVID-QU-Ex Dataset, for different purposes. MNIST is a popular dataset for training and testing image-based classification algorithms [51]. It consists of 60,000 training examples and 10,000 testing examples, each of which is a 28$\times$28 grayscale image labelled from 0 to 9. It is a widely used benchmark to evaluate the performance of various machine learning and computer vision models and is used as a pre-trained set for other image-based datasets.

The COVID-QU-Ex dataset, created by researchers at Qatar University, is a collection of 33,920 chest X-ray (CXR) images, including 11,956 COVID-19 images, 11,263 non-COVID infections (viral or bacterial pneumonia), and 10,701 standard images [34, 52]. The dataset also provides ground truth lung segmentation masks for the entire dataset and 2,913 COVID-19 infection segmentation masks from the QaTaCov project.

### Performance matrix’s

Precision, recall, F-measure, and confusion matrix are all measures of accuracy used to evaluate the performance of machine learning models.

Precision is a measure of how accurate the predictions of a model are. It is calculated as the ratio of accurate optimistic predictions (TP) to the sum of true positive and false positive predictions (TP + FP). A high precision value indicates that the model rarely predicts the wrong class.

The recall is a measure of how complete a model’s predictions are. It is calculated as the ratio of true positive predictions (TP) to the sum of true positive and false negative predictions (TP + FN). A high recall value indicates that the model rarely misses the correct class.

F-measure combines Precision and Recall into a single metric by computing the harmonic mean of Precision and Recall. It is calculated as the harmonic mean of Precision and Recall (2$\times$Precision$\times$Recall / (Precision + Recall)). A high F-value indicates that the model has a good balance between Precision and Recall.

The confusion matrix is a table that shows how the model classifies every possible combination of classes. It is used to calculate precision and recall. The confusion matrix is a visual representation of the model’s performance and can be used to identify areas for improvement.

### Analysis

 Recent advances in machine learning have enabled the development of models with improved performance. This research explores techniques to further enhance model performance, such as model training, evaluation, NSGA-II entropy-based sampling and training with adversarial examples. The use of multiple datasets in training models is a common practice, as it provides the model with greater generalizability. By using three datasets (training, validation and testing), the model can be prevented from overfitting to a particular dataset. Model training involves training the model on the dataset to achieve the desired performance. Evaluation measures the accuracy of the trained model by using performance metrics such as accuracy, precision and recall. NSGA-II entropy-based sampling is a technique that identifies a subset of data samples to maximize the entropy of a given dataset, which can improve the model's generalization. Training with adversarial examples is a technique to improve the robustness and generalization of a model by including negative examples in the training set. These are samples designed to be incorrectly classified by the model, which can reveal potential weaknesses and improve its performance. Finally, testing the model with the test set gives us an accurate measure of how the model performs on unseen data, which is essential for evaluating the quality of the uncertain samples created. To evaluate the impact of these techniques on model performance, experiments were conducted on public datasets. The results show that the use of these techniques leads to an increase in model performance. Moreover, different combinations of the techniques result in different performance improvements. Overall, the results of this research demonstrate that model training, evaluation, NSGA-II entropy-based sampling and training with adversarial examples are effective techniques for improving machine learning models’ performance. Further research is needed to explore the potential of these techniques for other application domains.

To evaluate the impact of these techniques on model performance, experiments were conducted on public datasets. The results of these experiments show that the use of these techniques leads to an increase in model performance. In addition, using different combinations of these techniques leads to different performance improvements. Overall, the results of this research show that model training, evaluation, NSGA-II entropy-based sampling, and training with adversarial examples are effective techniques for improving the performance of machine learning models. Further research is needed to explore the potential of these techniques for other application domains.

### MNIST data

**Fig. 2:**
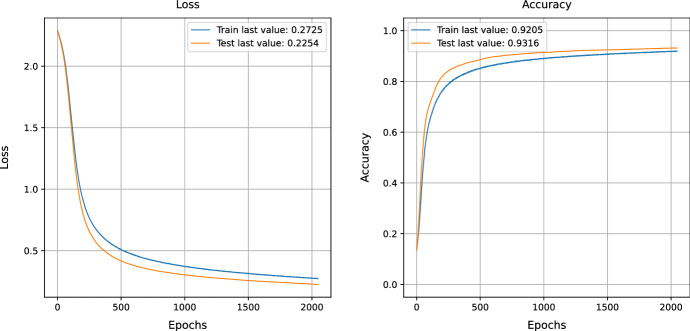
MNIST data training and evaluation of loss function before NSGA-II uncertain sample generation

Training the MNIST data and evaluating the loss function is one way to measure how well a model can learn the data, as mentioned in Fig. 2. The training loss of 0.27 indicates that the model can learn the data set accurately. The test loss of 0.22 indicates that the model can generalize the training data and accurately predict new data. The training accuracy of 0.92 and test accuracy of 0.9316 indicate that the model can accurately predict the effects of the data set. This shows that the model performs well and can accurately learn and predict the dataset.

**Fig. 3:**
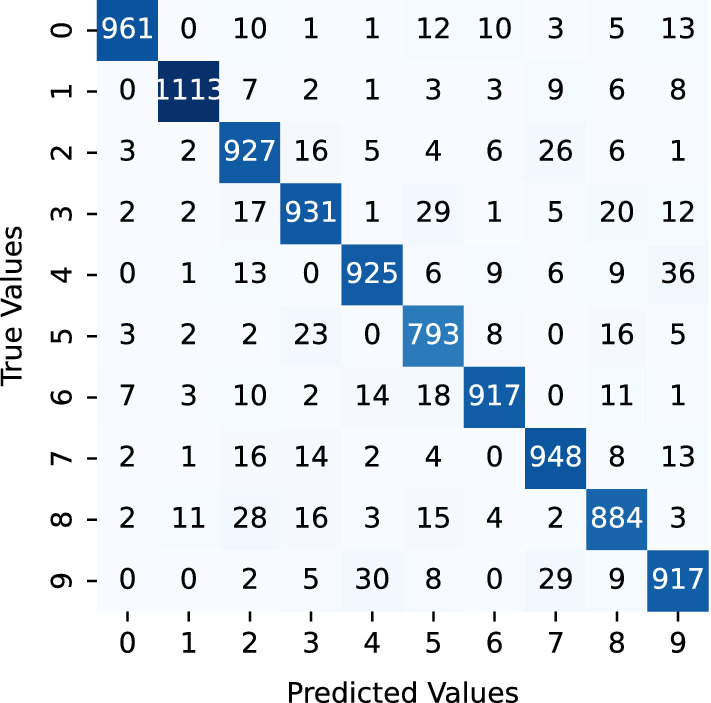
MNIST data confusion matrix before NSGA-II uncertain sample generation

 The Confusion Matrix of MNIST data prior to the application of NSGA-II uncertain sample generation is a great tool for visualizing the accuracy of a classifier's performance. It demonstrates how frequently the classifier correctly identified each class in the dataset. The matrix is divided into three distinct sections - a dark blue area representing the most accurately identified classes, a light blue area signifying classes that were correctly identified only in some cases, and a white area for the most misclassified classes. The dark blue area indicates the classes that were identified correctly the majority of times, while the light blue area indicates the classes that were correctly identified only occasionally. This instrument helps to identify classes that are misclassified and therefore, increases the accuracy of the classifier (Fig. 3).

**Fig. 4:**
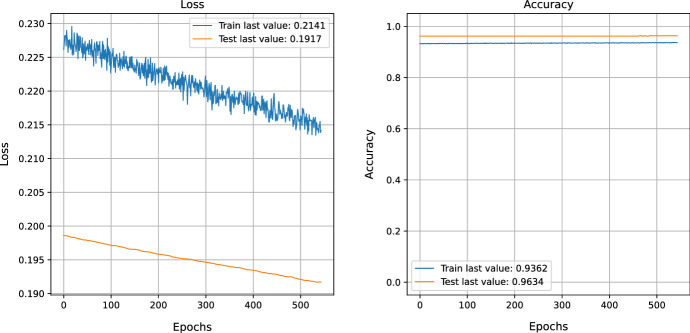
MNIST data training and evaluation of loss function After NSGA-II uncertain sample generation

**Fig. 5:**
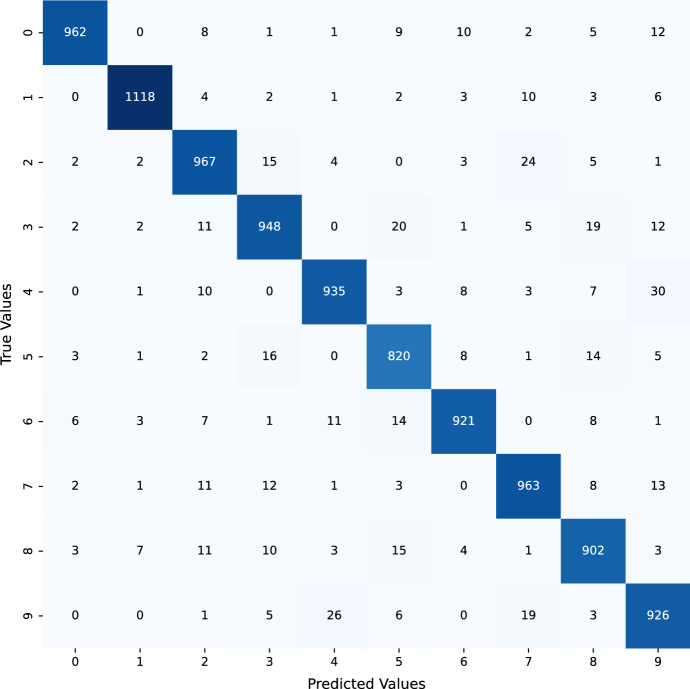
MNIST data confusion matrix after NSGA-II uncertain sample generation

**Fig. 6:**
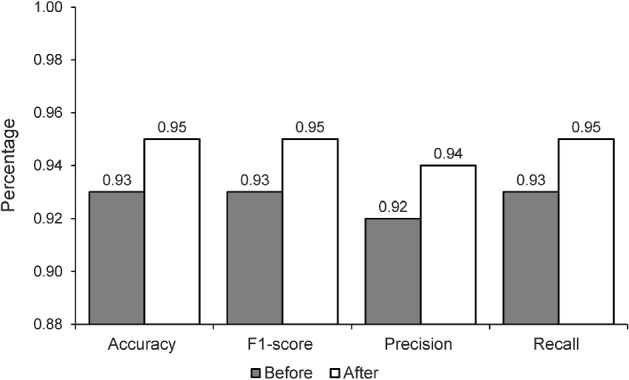
The comparison of the performance before and after the implementation of the proposed NSGA-based uncertainty samples generations

Training and evaluation of a loss function using the MNIST data and the NSGA-II algorithm for generating uncertain samples. After training, the results show a training accuracy of 0.93, a test accuracy of 0.96, a training loss of 0.21, and a test loss of 0.19, as mentioned in Fig. 4. This paper provides an in-depth look into the evolution of losses and accuracy when a model is trained with an adversarial test injection dataset. This evaluation is of great importance to assess the effectiveness of the model and its resistance to adversarial attacks. To comprehend the progression of losses and accuracy, we have analyzed the training methods to identify any potential vulnerabilities or imperfections that malicious attackers could take advantage of. The results demonstrate that we can modify the training methods to enhance the performance of the model against adversarial attacks by understanding the evolution of losses and accuracy. We can also recognize design flaws in the model that could be explored by malicious attackers. Figures  4 and 5 show that the accuracy improves as the losses decrease. Figure 6 demonstrates the precision of adversarial training, further demonstrating the performance metrics that develop with each training cycle. It clearly illustrates the decrease in uncertainty before and after the training process with the adversarial inputs.

The proposed technique works by augmenting the given dataset with unwanted inputs generated from the noise budget. Upon augmenting the dataset with these inputs, the model is retrained. It has been observed that the model's performance tends to worsen following the initial training with adverse inputs. However, the more training iterations that are performed, the more robust the model becomes, and the lower the uncertainty becomes. This is shown in Fig. 6, we observe a decrease in uncertainty in the model after it has been trained with unpredictable inputs. This technique can be particularly beneficial in cases where the model is interacting with the real world, as the data can be highly unpredictable and the model needs to be able to adapt accordingly. By training with unpredictable inputs, the model is able to better cope with changes in the data that it may not have seen before. Furthermore, this technique helps to reduce the risk of overfitting and improves the overall accuracy of the model. Not only does this technique make the model more robust and resilient, but it can also be used to detect and prevent malicious attacks on the model as it is designed to detect and correct any errors in the data.

.

**Fig. 7:**
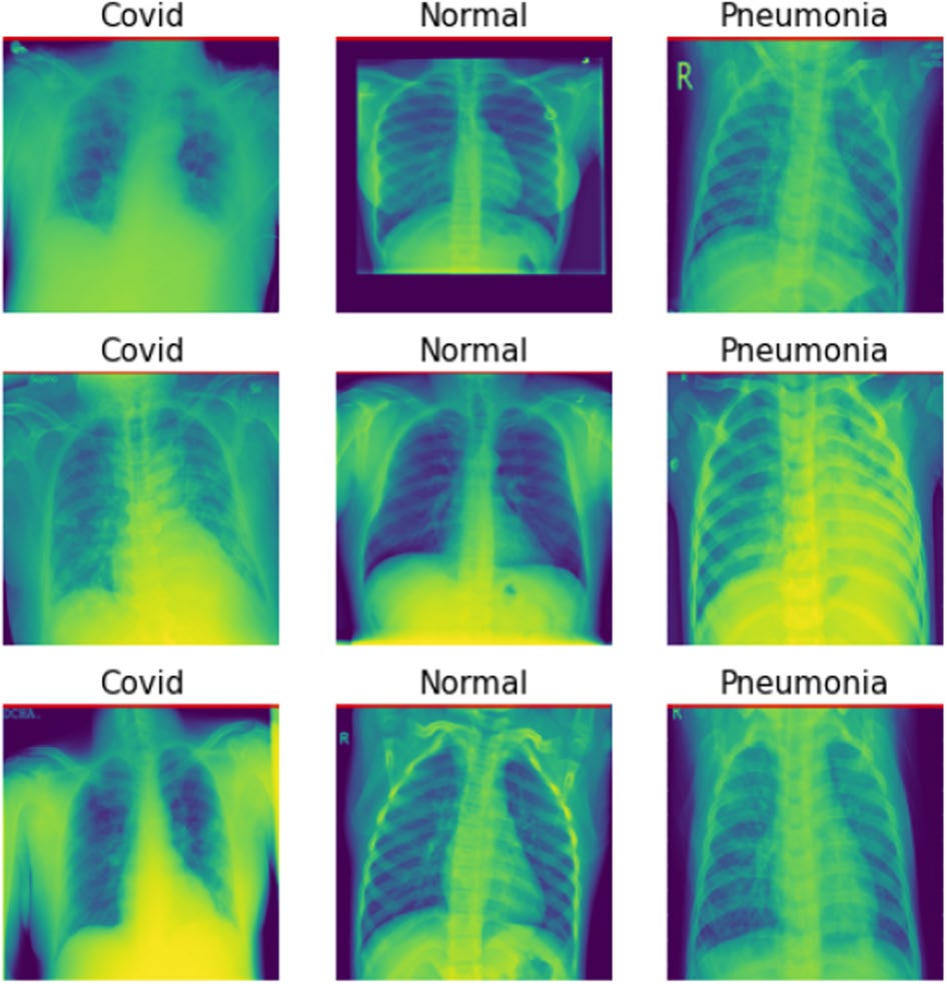
COVID data training class samples

### COVID-QU-Ex dataset

As shown in Fig. 7, COVID-19 has a highly contagious infection caused by the novel SARS-CoV-2 virus. It has had a drastic global impact, resulting in tens of millions of cases and hundreds of thousands of fatalities. Consequently, effective and dependable techniques for detecting and tracking the disease are urgently required. One approach to creating these technologies is to apply machine learning algorithms to patient data. To this end, researchers must amass and classify data from individuals with COVID-19 as well as pneumonia-related conditions. This data can then be used to create a dataset for training the algorithm.

An effective method for constructing a training set is to incorporate pixel density to distinguish between COVID-19, normal, and pneumonia patients. Pixel density is the ratio of pixels to the area of an image. By analyzing pixel density, researchers can determine which areas of an image are more affected by the disease. For instance, a higher lung pixel density may signify a more severe case of pneumonia. Incorporating pixel density into training sets is advantageous for various machine learning algorithms.

The NASGA-II Entropy-Based Sampling Technique is an evolutionary algorithm used to find the optimal solution for a given optimization problem. To evaluate this technique, a combination of pixel-as-image features and no-transfer learning method is used. Pixel image features are a technique for extracting useful information from images, such as shapes, edges, textures, and other features, by extracting pixel values from the image. No-transfer learning, on the other hand, is a type of machine learning that does not involve transferring knowledge from one domain to another. It relies solely on available data for learning and does not use prior knowledge or experience from other domains. The combination of these two techniques can be used to determine the optimal solution to a given optimization problem. In addition, undersampling is a technique used to balance a highly imbalanced dataset by randomly selecting a subset of the majority class and removing it from the dataset, thereby approximating the number of observations from the minority class. Care must be taken to ensure that the removed data is optional to the analysis. This technique is a valuable tool for balancing datasets when other techniques may be challenging.

**Fig. 8:**
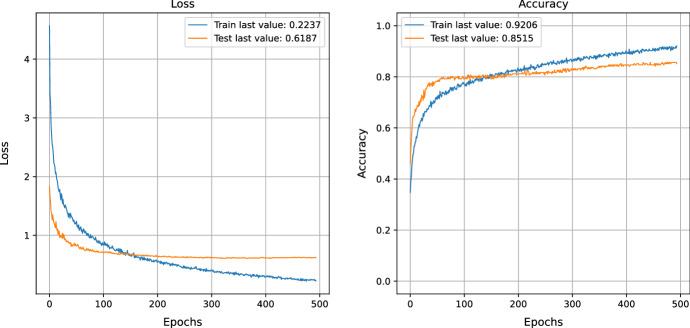
COVID data training and evaluation of loss function BEFORE NSGA-II uncertain sample generation

 As can be seen in Fig. 8, this case demonstrates that the model is performing well, as indicated by the training loss of 0.22 and the training accuracy of 0.92. However, the test loss of 0.61 and the test accuracy of 0.85 suggest that the model does not generalize well to new data. This could be attributed to the phenomenon of overfitting, wherein the model is too closely fitted to the training data and fails to generalize to unseen data.

**Fig. 9:**
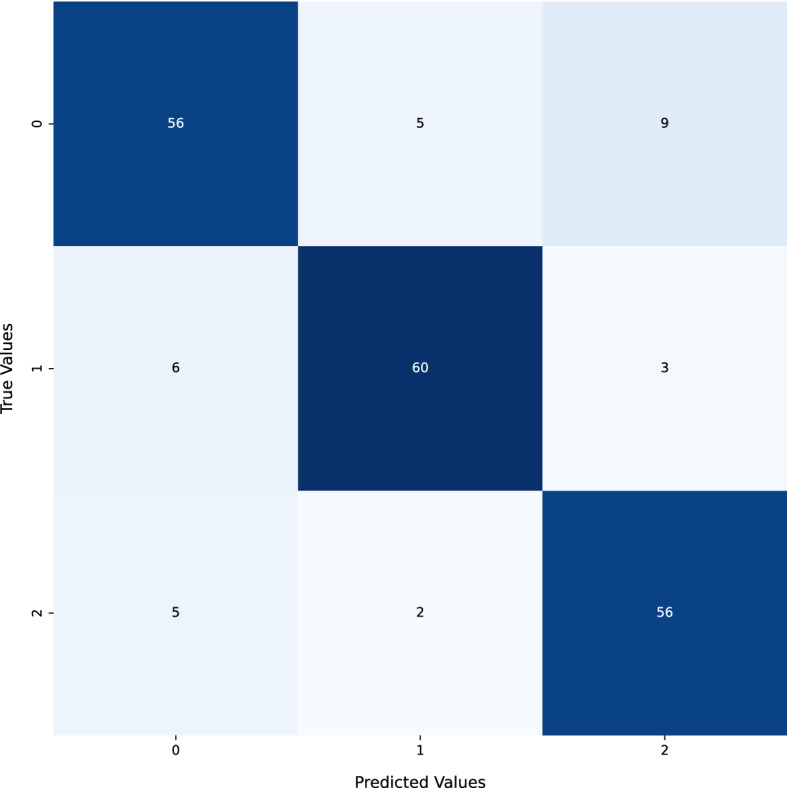
COVID data confusion matrix before NSGA-II uncertain sample generation

Unfortunately, the COVID data confusion matrix did not perform well, as shown in Fig. 9. Due to the highly complex nature of the data, it is difficult to make any precise predictions. To ensure accuracy, it is essential to create models that can incorporate new data and information as it becomes available.

**Fig. 10:**
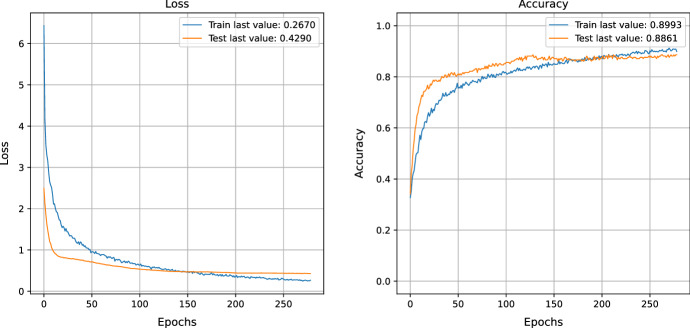
COVID data training and evaluation of loss function AFTER NSGA-II uncertain sample generation

NSGA-II is a multi-objective optimization technique used to generate diverse samples from a training dataset to prevent overfitting and improve a model's generalization. This method has been shown to yield a training loss of 0.26, a testing loss of 0.42, a training accuracy of 0.89, and a test accuracy of 0.88, as mentioned in Fig. 10. This implies that the model is able to learn more efficiently by creating unknown samples and implementing its knowledge towards previously unseen data.

Generating uncertain samples can enhance the model's learning process significantly as it is exposed to a greater number of data points and can recognize data patterns more precisely. This also improves the model's ability to generalize, allowing it to detect patterns in data that it has not encountered previously.

By utilizing NSGA-II, the impressive performance metrics of 0.26 training loss, 0.42 testing loss, 0.89 training accuracy, and 0.88 testing accuracy demonstrate that this approach is an effective way of enhancing the model's performance. This further highlights how the production of uncertain samples can boost the accuracy and extend the generalizability of the model.

**Fig. 11:**
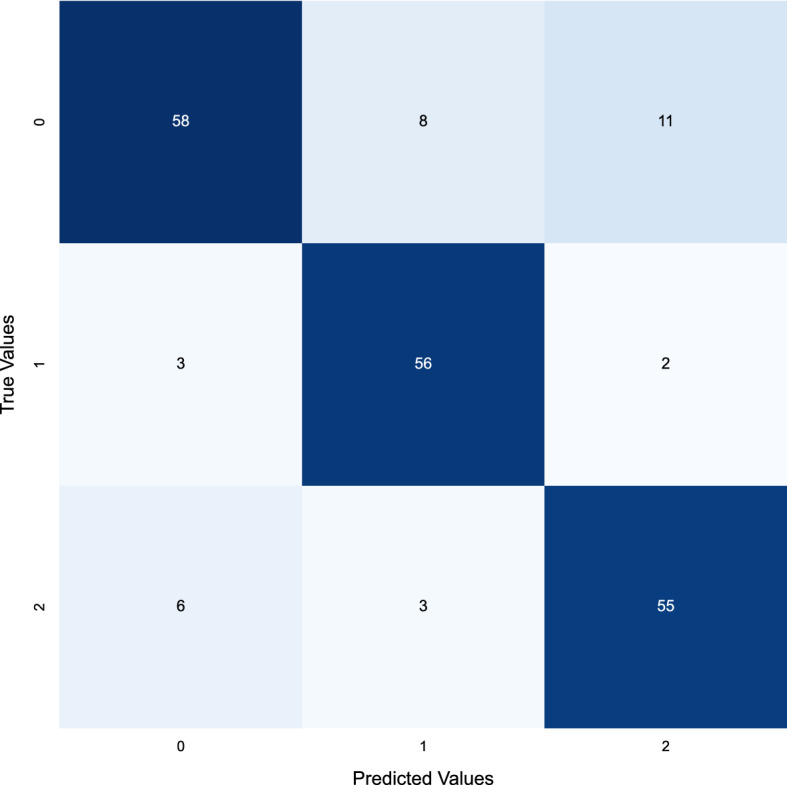
COVID confusion matrix after NSGA-II uncertain sample generation

The accuracy of the matrix can be improved by a technique known as NSGA-II uncertain sample generation, as mentioned in Fig. 11. This method of Deep Learning Uncertainty Quantification creates a collection of uncertain samples from a given data set. These samples are then utilized to train a model, which is then employed to evaluate the precision of the confusion matrix. This technique can enhance the accuracy of the confusion matrix by decreasing the number of false positives and negatives.

**Fig. 12:**
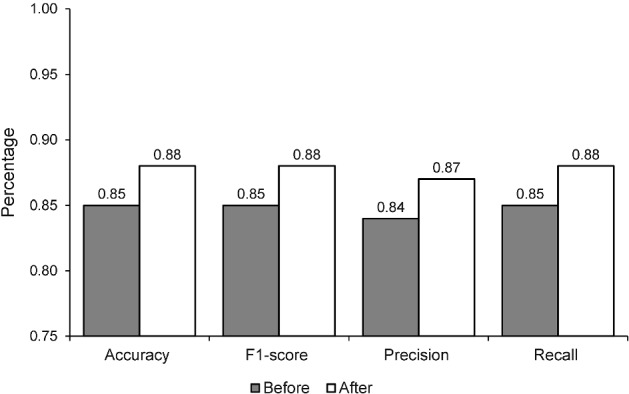
The confusion matrix of the COVID dataset after using the NSGA-based uncertainty sample generations

Overall, using NSGA-II to generate uncertain samples can help improve the accuracy of the COVID confusion matrix, as mentioned in Fig. 12. Deep learning uncertainty quantification can reduce the occurrence of both false positives and false negatives, thereby helping to reduce the transmission of the virus and improve the accuracy of diagnostic tests.

## Conclusions

Adversarial attacks are a major concern in ML due to their capacity to manipulate model inputs and cause the model to make erroneous predictions. In this study, we research the utilization of UQ in medical imaging and diagnosis. UQ can be employed to gauge the risk of misdiagnosis and enhance the accuracy of ML models, medical diagnoses, and healthcare systems. We investigate and compare UQ measures through transfer learning and uncertainty analysis in the COVID Radiography Database to evaluate the risk of misdiagnosis. We also present a robust DL model that can manage highly uncertain inputs. Our proposed approach is divided into three stages: Generation of a dataset, construction of a dropout-based neural network, and retraining of the model with highly uncertain inputs. To gauge the effectiveness of our approach, we computed the entropy values for all examples and selected an example with the highest entropy value as a candidate. The outcomes of this study suggest that UQ can be utilized to refine the classification of radiographic images and to assess the risk of misdiagnosis of medical images. Finally, we address the implications of these UQ applications for the optimization and development of healthcare systems. In this paper, we also present a research project that uses the NGSA-II entropy-based sampling-based optimization model to optimize a machine learning model. This approach provides precise results yet requires a longer execution time. To reduce this time and improve the optimization process, the researchers explored uncertainty maximization based on the loss function of DL, which offers comparable results in a shorter period. To further refine the optimization process, this paper also proposes to investigate uncertainty maximization based on the DL loss function and Kernel Density Estimation (KDE). The usage of these strategies could provide accurate results quickly, reduce the uncertainty associated with the model, and deter overfitting, eventually leading to better performance.

## Data Availability

Two datasets (MINIST^1^ and COVID-QU-Ex^2^) are available on the public repositories.
